# Evaluating the *MT-CYB* and *MT-ATP6* variations in COVID-19 patients: A case-control study

**DOI:** 10.1371/journal.pone.0329866

**Published:** 2025-08-21

**Authors:** Gazi Nurun Nahar Sultana, Md Zahid Hasan, Arindita Das, Robin Sarker, Hossain Uddin Shekhar, Rokeya Begum

**Affiliations:** 1 Centre for Advanced Research in Sciences (CARS), University of Dhaka, Dhaka, Bangladesh; 2 Department of Biochemistry and Molecular Biology, University of Dhaka, Dhaka, Bangladesh; 3 Department of Genetic Engineering and Biotechnology, University of Dhaka, Dhaka, Bangladesh; Central University of Kerala, INDIA

## Abstract

The complications and lingering effects of COVID-19 have caused a global health crisis, prompting intense investigations into the mechanism by which SARS-CoV-2 causes the disease. The majority of symptoms associated with this infectious disease are linked to mitochondrial dysfunction, and recent studies indicate that SARS-CoV-2 can impair host mitochondrial function. This study aims to investigate mutations in the *MT-CYB* and *MT-ATP6* genes of mtDNA in COVID-19 patients and their association with disease outcomes. Out of 110 individuals enrolled, 30 were diagnosed with COVID-19, while the remaining 80 were healthy. Following DNA isolation from the blood samples, the *MT-CYB* and *MT-ATP6* genes were amplified through PCR, purified, and sequenced using Sanger sequencing. In the *MT-CYB* gene, 9 distinct mutations were found. Among these, novel mutation m.14942A > C was more prevalent in COVID-19-positive individuals than COVID-19 negative controls (p = 0.012 < 0.05) and exhibited a significant correlation with the disease as OR (95% CI) = 19.75 (2.264–172.246). According to *in-silico* analyses, this mutation is deemed deleterious and decreases the stability of the CYB protein. In case of the *MT-ATP6* gene, among the identified 8 mutations, the m.8744T > G mutation was higher in COVID-19-positive individuals than healthy controls (p = 0.009 < 0.05) and showed a significant correlation with the disease: OR (95% CI) = 24.04 (2.81–205.62). According to *in-silico* analyses, this mutation is found to be pathogenic and reduces the stability of the ATP6 protein. In conclusion, one novel mtDNA mutation was identified in this study, and some of the mutations identified in the *MT-CYB* as well as *MT-ATP6* genes, including the novel mutation, are relatively common in the COVID-19 patient group. Moreover, the findings demonstrated that these mutations may contribute to the pathogenesis of COVID-19.

## 1. Introduction

The COVID-19 pandemic, caused by the severe acute respiratory syndrome coronavirus 2 (SARS-CoV-2), has created an unprecedented global health crisis, impacting millions of individuals worldwide [1]. First discovered in Wuhan, China, in December 2019, this highly contagious virus rapidly spread and affected people on every continent [2]. The illness can cause a wide range of symptoms, from a common cold or moderate respiratory distress to severe pneumonia. In the worst situations, it may lead to organ failure [3]. Millions of people have already died as a result of the COVID-19 epidemic, which began in 2020 and evolved into a worldwide pandemic [4]. It has had an unparalleled effect on public health, the economy, and social life, and other sectors [5].

The SARS-CoV-2 viral genome and proteins such as ORF9b, ORF3a, M, and NSP8 have been detected in mitochondria [6]. Proteomic data and functional assays support that ORF9b and ORF3a proteins of SARS-CoV-2 modulate and subvert mitochondrial function by disrupting membrane integrity and mitochondrial antiviral signaling (MAVS) [7]. Recent studies suggest that mitochondrial residency may be necessary for the formation of double-membrane vesicles (DMVs), which are essential for the unchecked replication of coronavirus as it eludes cellular defenses [8]. Based on the provided data, SARS-CoV-2, upon gaining control of mitochondria, enlists the help of these organelles to facilitate its virulence and transmissibility. These phenomena are known as mitochondrial hijacking by the virus [9]. Exploitation of mitochondria by SARS-CoV-2 leads to mitochondrial dysfunction, including disruption of membrane integrity, increased production of reactive oxygen species (ROS), and impaired oxidative phosphorylation [10]. Such stressors are known to damage mitochondrial DNA (mtDNA), potentially leading to mutations.

Human mtDNA is a double-stranded DNA molecule that encodes 13 structural peptide subunits of the oxidative phosphorylation system, along with 24 RNA molecules essential for protein synthesis within mitochondria [11]. Mitochondrial DNA is exposed to a mutation rate 100 times higher than that of the nuclear genome because the mtDNA repair mechanisms are less robust (more error-prone) than nuclear DNA repair systems [12]. Human mature erythrocytes lack mitochondria, while every other human cell contains them [13]. Mitochondria are essential parts of intermediate metabolism in several cellular metabolic pathways, such as fatty acid oxidation, oxidative phosphorylation, the Krebs cycle, the urea cycle, gluconeogenesis, and ketogenesis [11]. A disruption of the fundamental bioenergetic, antioxidant, and regulatory functions of the mitochondria is usually indicative of mitochondrial malfunction [14].

The mitochondrial cytochrome b (*MT-CYB*) gene encodes a subunit of respiratory Complex III that catalyzes the transfer of electrons from ubiquinol (reduced Coenzyme Q10) to cytochrome c [15]. *MT-ATP6* is a mitochondrial gene with the full name ‘mitochondrially encoded ATP synthase membrane subunit 6’ that encodes the ATP synthase F_O_ subunit ‘a’ or ‘6’ [16]. This subunit is a crucial component of proton channels and contributes to the oxidative phosphorylation process. Products encoded by these genes are shown to be important in several cellular events, including the induction of inflammatory events in lungs and hepatocytes, apoptosis, renal fibrosis, and chronic kidney disease (CKD) [16].

SARS-CoV-2 infection can lead to mtDNA mutations and mitochondrial dysfunction, which may contribute to the pathophysiology of COVID-19 and its long-term sequelae, particularly in tissues with high mitochondrial dependence. However, the available information on this topic is limited, especially in the case of Bangladeshi COVID-19 patients. That’s why the study aimed to identify mutations in the *MT-CYB* and *MT-ATP6* genes of mtDNA in COVID-19 patients and healthy individuals, assess the association of identified mutations with disease outcomes, and evaluate the pathogenicity of mutations found in COVID-19 patients compared to healthy controls. To be precise, the research questions are whether mutations in *MT-CYB* and *MT-ATP6* are more prevalent or pathogenic in Bangladeshi COVID-19 patients than in healthy individuals, and whether these mutations are associated with disease severity or clinical outcomes.

## 2. Materials and methods

### 2.1 Blood collection and preservation

With ethical permission (Ref No. 127/Biol.Scs.) from the institutional ethical review committee, this study comprised 80 healthy participants from Bangladesh and 30 individuals previously infected with SARS-CoV-2. With blood collection taking only a few minutes for each participant, they were recruited and provided blood samples on the same day. Blood collection for this study was conducted between November 4, 2020, and February 2, 2021. Written informed consent was obtained from all the participants, and their age was more than 18 years, i.e., there were no minor participants included in this study. Venous blood of 5 mL was collected in an EDTA-coated vacutainer tube with the help of expert phlebotomists from each study participant with informed consent. The samples were then carried in a sample carrier, while maintaining a temperature of 2–8 °C.

### 2.2 Extraction of DNA from blood samples and quantification of DNA

To isolate the total genomic DNA, the Wizard® Genomic DNA Purification Kit by Promega was used. Utilizing a NanoDrop® spectrophotometer, DNA was quantified, and its purity was assessed. To visualize the DNA, a mixture of 2 μL of the extracted DNA solution and 2 μL of 1 × loading dye was added to the 1.5% (w/v) agarose gel wells, and agarose gel electrophoresis was performed. Quantity One software was used to observe the DNA bands in the gel.

### 2.3 Amplification by PCR and PCR product purification

Two sets of primer pairs, 22F (5’-AACTGCAGTCATCTCCGGTTTACAAGA-3’) and 22R (5’-GGAATTCATCTCTCCGGTTTACAAGA-3’) as well as 13F (5’-TTTCCCCCTCTATTGATCCC-3’) and 13R (5’-GTGGCCTTGGTATGTGCTTT-3’), were chosen by literature mining to amplify the *MT-CYB* and *MT-ATP6* genes, respectively [17]. The polymerase chain reaction (PCR) master mix was prepared by thawing and spinning the components (Table 1). In each PCR tube, 30 ng of template DNA (1 μL) was mixed with 9 μL of PCR master mix. Experiments were conducted using the Applied Biosystems’ ProFlex PCR system. For amplification of the *MT-ATP6* gene, the cycling conditions included initial denaturation at 95 °C for 5 minutes, followed by 35 cycles of denaturation at 94 °C for 30 seconds, annealing at 58 °C for 30 seconds, and elongation at 72 °C for 2 minutes. The final extension step was 7 minutes at 72 °C. To amplify the *MT-CYB* gene, similar cycling conditions were applied, but the duration of the final extension was 5 minutes at 72 °C. Successful amplification was confirmed by agarose gel electrophoresis. Further, the PCR products were purified using ExoSAP-IT™ PCR Product Cleanup kits by following the manufacturer’s protocol.

**Table 1:** PCR master mix components for a total of 10 μL reaction volume.

Components	Amount per Reaction	
	For *MT-CYB*	For *MT-ATP6*
10 × PCR buffer	1.00 μL	1.00 μL
MgC1_2_ (25 mM)	0.60 μL	0.60 μL
Deoxynucleotide triphosphates (dNTPs) (10 mM)	0.80 μL	0.80 μL
Forward primer (3.2 pM)	0.15 μL	0.08 μL
Reverse primer (3.2 pM)	0.15 μL	0.08 μL
Taq DNA polymerase (1 U)	0.15 μL	0.30 μL
Nuclease free H_2_O	6.65 μL	6.14 μL

### 2.4 Cycle sequencing and capillary electrophoresis

PCR amplicons for *MT-CYB* and *MT-ATP6* were sequenced using the Applied Biosystems BigDye® Terminator v3.1 kit. Applied cycling conditions of the sequencing PCR consisted of an initial denaturation period of 3 minutes at 95 °C; followed by 30 cycles at 94 °C for 10 seconds, 55 °C for 5 seconds, and 60 °C for 4 minutes. Subsequently, the sequencing PCR products were purified through ethanol/EDTA/sodium acetate precipitation. The resulting products were analyzed with capillary electrophoresis in the SeqStudio™ Genetic Analyzer.

### 2.5 Mutation detection and in-silico analyses

Using Geneious Prime software V202, the mtDNA sequences were aligned and compared with the revised Cambridge Reference Sequence (rCRS) (NCBI Reference Sequence: NC_012920.1), and genetic variations were found. *In-silico* analyses were done to enhance our understanding of genetic variations. This involved predicting the pathogenicity of non-synonymous SNPs (nsSNPs) and assessing their impact on protein stability with the help of multiple tools. A majority-vote approach was employed, whereby a variant was considered potentially pathogenic or structurally destabilizing if the majority of tools predicted it as such. This strategy was adopted to reduce bias from any single tool and to emphasize consensus across diverse predictive algorithms. Besides, to analyze the surface accessibility of nsSNPs, NetsurfP-2.0 was utilized, and deleterious effects on protein structure were evaluated using the HOPE server. Additionally, 3D structure modeling of mutated proteins was performed by DynaMut, providing data on their structural alterations.

### 2.6 Statistical analysis

Graphs and charts were generated using R Studio and Microsoft Excel. The association of mtDNA variants with disease was explored using SNPStats. This tool provided allele and genotype frequencies, descriptive statistics, and percentages for categorical variables. Moreover, it offered insights into p-values, odds ratios (OR), mean differences, and 95% confidence intervals from multivariate logistic regression analyses. The values were considered significantly different at p-value < 0.05.

## 3. Results

### 3.1 Demographic characteristics of the case and control

The nationality of the study population is Bangladeshi, and they are from Bengali ethnic groups. The average age of patients was 37.77 ± 13.92 years, while the average age of healthy participants was 31.71 ± 12.05 years (S1 Table). Even though the ages of the two study groups showed no substantial difference, the slight difference was statistically significant (p-value = 0.041 < 0.05). Among the patients, 63.33% were male and 36.67% were female, whereas 51.25% were male and 48.75% were female among healthy individuals (S1 Table). There was no statistically significant difference in sex distribution between the patient and control groups, as determined by the Chi-square test (p-value = 0.297 > 0.05). These findings indicate that sex is unlikely to be a confounding factor in this study, whereas age might be. That is why multivariate analyses were performed during the association study between variants and disease outcome to address any potential confounding effects from age.

### 3.2 Single nucleotide variations found within the *MT-CYB* and *MT-ATP6* gene regions

The nucleotide sequences in the *MT-CYB* gene region have been found to have 9 different single nucleotide variations shown in Table 2. Out of 9 variations, 5 mutations were confirmed to be specific to the patients, and the remaining 4 were present in both participant groups. In this investigation, 3 variants (m.14942A > C, m.15301G > A, m.15315C > T) in total were found to be non-synonymous SNPs (nsSNPs). In the table, all other nucleotide changes leading to identical amino acids (e.g., Thr to Thr, Gly to Gly, or Phe to Phe) are considered synonymous.

**Table 2:** Variants detected in the *MT-CYB* gene of the study participants with their frequencies.

mtDNA Position	Nucleotide Change	Amino Acid Change	Frequency in Sample (n = 30)	Frequency in Controls (n = 80)	p-Value
14942	A → C	Ile66Leu	20% (6/30)	0% (0/80)	0.01165
14956	T → C	Thr70Thr	3.33% (1/30)	0% (0/80)	0.325582
15043	G → A	Gly99Gly	80% (24/30)	63% (50/80)	0.062096
15049	C → T	Gly101Gly	3.33% (1/30)	0% (0/80)	0.325582
15295	C → T	Phe183Phe	3.33% (1/30)	0% (0/80)	0.325582
15301	G → A	Leu185Leu	70% (21/30)	52.85% (42/80)	0.091667
15315	C → T	Ala190Val	3.33% (1/30)	0% (0/80)	0.325582
15326	A → G	Thr194Ala	100% (29/30)	100% (80/80)	0.325582
15346	G → A	Leu200Leu	3.33% (1/30)	0% (0/80)	0.325582

The nucleotide sequences in the *MT-ATP6* region have been found to have 8 different single nucleotide variations, shown in Table 3. Among these, 1 variation was specific to the patients, and the rest 7 mutations were present in both participant groups. In this investigation, 4 variants (m.8701A > G, m.8744T > G, m.8794C > T, and m.8860A > G) in total were found to be nsSNPs.

**Table 3:** Variants detected in the *MT-ATP6* gene of study participants with their frequencies.

mtDNA Position	Nucleotide Change	Amino Acid Change	Frequency in Sample (n = 30)	Frequency in Controls (n = 80)	p-Value
8701	A → G	Thr59Ala	70% (21/30)	41.25% (33/80)	0.0064
8744	T → G	Val73Gly	23% (7/30)	1.25% (1/80)	0.009
8794	C → T	His90Tyr	3.33% (1/30)	5% (4/80)	0.6885
8860	A → G	Thr112Ala	100% (30/30)	100% (80/80)	--
8911	T → C	Leu129Leu	6.66% (2/30)	0% (0/80)	0.1607
8928	T → C	Pro134Pro	3.33% (1/30)	1.25% (1/80)	0.5619
9003	C → T	Arg159Arg	13.33% (4/30)	6.25% (5/80)	0.3091
9004	C → T	Leu160Leu	13.33% (4/30)	5% (4/80)	0.1693

Along with the frequency seen in the research population, the global frequencies of all nsSNPs found in *MT-CYB* and *MT-ATP6* genes were determined from the MITOMAP and gnomAD3.1 databases. Patterns of frequency distribution with respect to the identified variants have been presented in Table 4. In the *MT-CYB* gene, one mutation m.14942A > C is previously unreported (novel) (S1 Fig). In the case of m.14942A > C in the *MT-CYB* gene and m.8744T > G in the *MT-ATP6* gene, the mutation frequency is significantly higher in patients than in the MITOMAP and gnomAD 3.1 databases.

**Table 4:** Frequency of the variants found in the Patients, MITOMAP, and gnomAD3.1.

Gene	Variations	Patients (%)	MITOMAP (%)	gnomAD 3.1 (%)
*MT-CYB*	m.14942A > C	20	0	0
*MT-CYB*	m.15315C > T	3.33	0.34	0.04
*MT-CYB*	m.15326A > G	100	98.605	99.342
*MT-ATP6*	m.8701A > G	70	31.225	30.319
*MT-ATP6*	m.8744T > G	23	0.002	0.004
*MT-ATP6*	m.8794C > T	3.33	2.859	4.75
*MT-ATP6*	m.8860A > G	100	98.522	99.381

### 3.3 Association of variants with disease outcome

**Fig 1:**
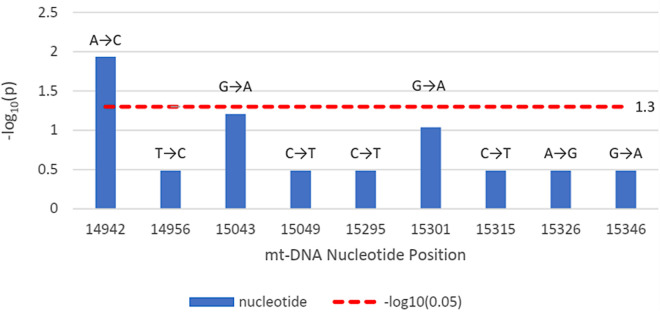
The bar plot illustrating the significance of the association between disease outcome and mutations within the *MT-CYB* (14747-15887 bp) gene. The y-axis indicates the –log_10_ of p-values, while the x-axis indicates nucleotide locations. The red dotted line denotes the –log_10_(0.05), i.e., significance level. Any points that are greater than or equal to the line are designated as statistically significant (p-value 0.05).

**Fig 2:**
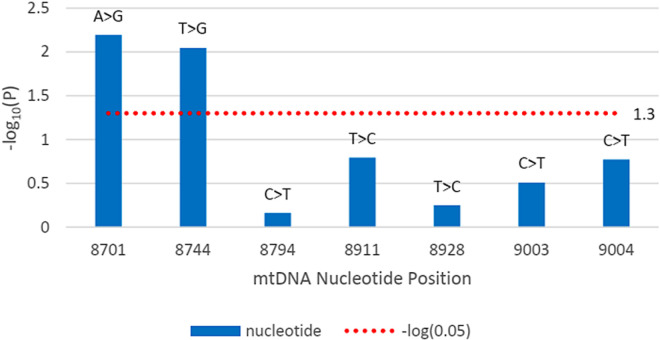
The bar plot illustrating the significance of the association between disease outcome and mutations within the *MT-ATP6* (8527–9207 bp) gene. The y-axis indicates the –log_10_ of p-values, while the x-axis indicates nucleotide locations. The red dotted line denotes the significance level, i.e., –log_10_(0.05). Any points that are greater than or equal to the line are designated as statistically significant (p-value 0.05).

The disease outcome was studied for all variations found within the *MT-CYB* and *MT-ATP6* genes. Fig 1 shows that in the case of the *MT-CYB* gene, m.14942A > C had a significant correlation (p = 0.012 < 0.05) with the likelihood of COVID infectivity (odds ratio = 19.75), as the -log_10_(p) value crosses the threshold value. In the case of *MT-ATP6*, m.8701A > G had a significant correlation (p = 0.006 < 0.05) with the likelihood of COVID-19 infectivity (odds ratio = 3.32), and m.8744T > G had a significant correlation (p = 0.009 < 0.05) with the likelihood of COVID-19 infectivity (odds ratio = 24.04), represented by Fig 2.

### 3.4 Determination of the pathogenicity of the nsSNPs

**Fig 3:**
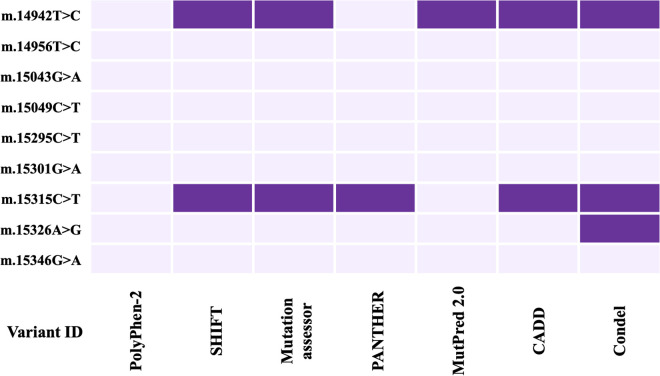
Heatmap of the prediction of deleterious impact on CYB protein due to nsSNPs using seven pathogenicity prediction tools (Polyphen-2, SIFT, Mutation assessor, PANTHER, Mutpred 2.0, CADD, and CONDEL). The purple color indicates a deleterious impact on protein.

**Fig 4:**
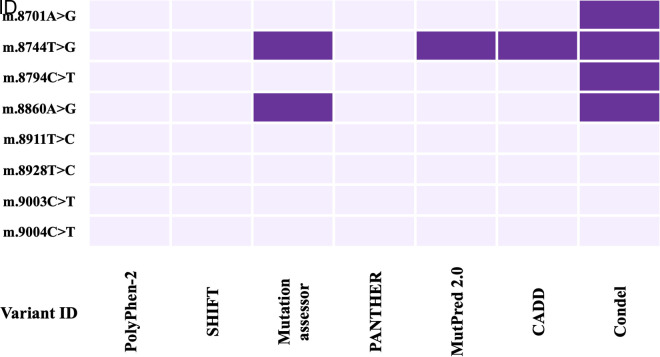
Heatmap of the prediction of deleterious impact on ATP6 protein due to nsSNPs using seven pathogenicity prediction tools (Polyphen-2, SIFT, Mutation assessor, PANTHER, Mutpred 2.0, CADD, and CONDEL). The purple color indicates a deleterious impact on protein.

Different web-based tools were developed using different algorithms and approaches to assess the severity of the pathogenicity of the identified nsSNPs. 7 tools named Polyphen-2, SHIFT, Mutation assessor, PANTHER, MutPred-2, CADD, and Condel were used to increase the robustness of the prediction. Mutations m.14942A > C and m.15315C > T in the *MT-CYB* gene were projected to be deleterious by 5 tools, the most of any of the 3 nsSNPs (Fig 3), and were identified only in the patients. In the *MT-ATP6* gene, the m.8744T > G mutation was projected to be deleterious by 4 tools, the most of any of the 4 nsSNPs (Fig 4), and was present more in the patients than healthy controls.

### 3.5 Determination of the link between nsSNPs and protein stability modification

**Fig 5:**
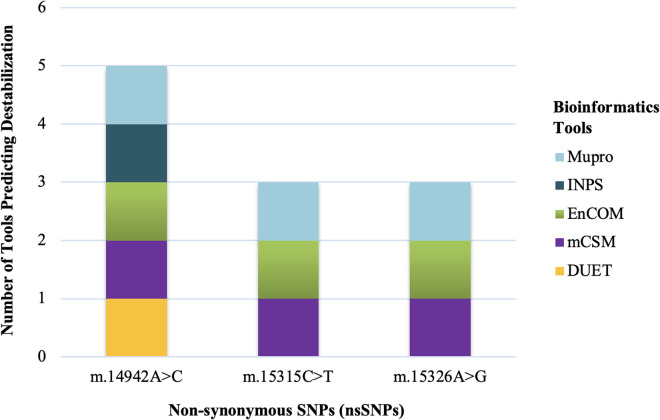
Stacked bar plot presenting the CYB protein destabilizing effect due to nsSNPs predicted by 5 different tools (Mupro, INPS, EnCOM, mCSM, and DUET).

Five tools, DUET, mCSM, EnCOM, INPS-MD, and MuPro, were used to predict the probable changes in protein stability resulting from amino acid alterations due to the presence of nsSNPs. All tools predicted the ΔΔG (kcal/mol) value for each mutated protein, indicating the difference in unfolding free energy between the wild type and mutant protein. Here, ΔΔG < 0 and ΔΔG > 0 were considered as decreased and increased stability, respectively. Firstly, nsSNPs in the *MT-CYB* gene were analyzed. m.14942A > C was predicted to be destabilizing by all tools. But m.15315C > T and m.15326A > G were predicted to be destabilizing by mCSM, EnCOM, and Mupro but stabilizing by DUET and INPS-MD (Fig 5).

**Fig 6:**
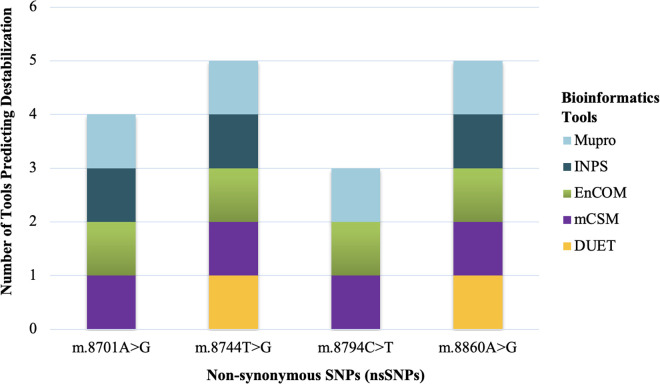
Stacked bar plot presenting the ATP6 protein destabilizing effect due to nsSNPs predicted by 5 different tools (Mupro, INPS, EnCOM, mCSM, and DUET).

Furthermore, nsSNPs in the *MT-ATP6* gene were analyzed; m.8744T > G and m.8860A > G were predicted to be destabilizing by all tools. However, m.8701A > G was predicted to be destabilizing by mCSM, EnCOM, INPS, and Mupro but stabilizing by DUET (Fig 6).

### 3.6 Surface accessibility of nsSNP sites

To find the surface accessibility of specific amino acid sites, NetSurfP 3.0 was utilized. The relative surface accessibility (RSA) value was used to measure surface accessibility; an amino acid was considered exposed to the surface if its RSA value was more than 25%. The position of the amino acid modification (hidden or surface exposed) on the protein was ascertained with the use of this prediction. m.14942A > C mutation was not exposed to the surface, but m.15315C > T and m.15326A > G in the *MT-CYB* gene were exposed to the surface according to NetsurfP 3.0 (S2 Fig). However, except for m.8860A > G, all nsSNPs in the *MT-ATP6* gene were exposed to the surface of the protein (S3 Fig).

### 3.7 nsSNPs and their effects on the function of CYB and ATP6 proteins

The properties of the changed amino acids caused by the nsSNPs were assessed based on the results of HOPE. Changes in hydrophobicity, size, and overall properties for nsSNPs in the *MT-CYB* and the *MT-ATP6* regions are noted in Table 5. It was expected that every nsSNP located in the *MT-CYB* gene would be involved in several activities, such as electron transfer activity, oxidoreductase activity, and reductase activity of ubiquinol-cytochrome-C and every nsSNP located in the *MT-ATP6* gene is involved in several activities, such as proton transmembrane transporter activity. Changes in these areas could therefore have an impact on how these pathways function.

**Table 5:** Prediction of structural effect in the CYB and ATP6 protein due to nsSNPs using the HOPE web tool.

Mutation	Altered Size	Altered Hydrophobicity	Significant Impacts
m.14942A > C	No Data	No Data	Located in a domain that is important for the activity and contact with another domain that is also important for the activity.
m.15315C > T	Bigger	Increased	Could affect the local stability and can affect contacts with the lipid membrane.
m.15326A > G	Smaller	Increased	Cause a space in the core of the protein leading to disturbance in correct folding.
m.8701A > G	Smaller	Increased	Located in a domain that is important for the activity and contact with another important domain.
m.8744T > G	Smaller	Increased	This mutation could affect multimer contacts. Glycine is very flexible and can disturb the required rigidity of the protein at this position.
m.8794C > T	Bigger	Increased	The mutation will cause a loss of hydrogen bonds in the core of the protein and disturb correct folding.
m.8860A > G	Smaller	Increased	The mutation will cause a loss of hydrogen bonds in the core of the protein and disturb correct folding.

### 3.8 Alterations of protein structure due to m.14942A>C mutation in the *MT-CYB* gene

**Fig 7:**
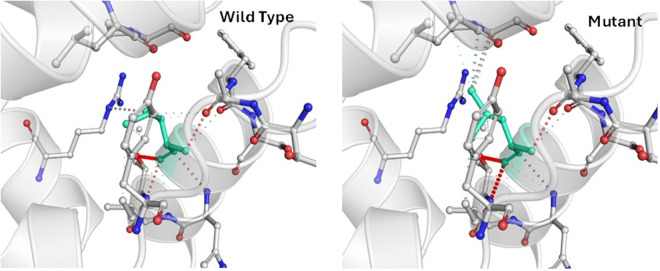
Interatomic interaction variations due to m.14942A > C mutation in the *MT-CYB* gene. Wild-type and mutant residues are colored light green and are also represented as sticks alongside the surrounding residues which are involved in any type of interaction.

Since m.14942A > C showed a significant correlation with disease outcome, and most of the tools predicted it to be deleterious. It might also decrease protein stability. The mutant residue is situated in a domain that is critical to the protein’s action and close to another equally crucial domain. Hence, this mutation could potentially disrupt the interaction between these domains, thus impacting the protein’s function. To determine the structural consequences of the altered amino acids, the protein sequence was retrieved from UniProtKB (P00156), and the protein’s wild-type and mutant three-dimensional structure was retrieved from the Dynamut webserver shown in Fig 7.

This mutation has created a distinct alteration in the protein’s interactions with adjacent amino acids. The loss of a hydrogen bond with the previous amino acid and the development of new hydrogen bonds lead to major changes in the protein’s structure. These modifications could have repercussions for the protein’s stability, function, and overall biological activity.

### 3.9 Alterations of protein structure due to m.8744T>G mutation in the *MT-ATP6* gene

As m.8744T > G showed the mutation of a valine into a glycine at position 73, which has a significant correlation with the disease outcome, and most tools identified it as deleterious. It might also decrease protein stability. The new residue, glycine, might be too tiny to make multimer interactions. Glycines are very flexible and can affect the essential stiffness of the protein at this location. The mutation introduces a less hydrophobic residue. Sometimes, hydrophobicity is vital for multimer interactions.

**Fig 8:**
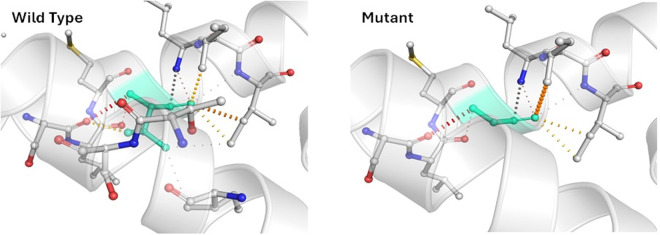
Interatomic interaction variations due to m.8744T > G mutation in the *MT-ATP6* gene. Wild-type and mutant residues are colored light green and are also represented as sticks alongside the surrounding residues which are involved in any type of interaction.

The mutant residue is located in a domain essential to the protein’s action and is near another equally crucial domain. Thus, this mutation could disrupt the interaction between these domains, thereby affecting the protein’s overall functionality. To determine the structural consequences of the altered amino acids, the protein sequence was retrieved from UniProtKB (P00846), and the protein’s wild-type and mutant three-dimensional structure was retrieved from the Dynamut webserver shown in Fig 8.

The mutation m.8744T > G substantially affected the interactions with surrounding amino acids in the protein structure. In comparison to the wild-type structure, the mutant form displayed a considerable decline in contacts and exhibited loss of a hydrogen bond with the previous amino acid and the creation of new hydrogen bonds. This mutation signifies a potential disturbance in the protein’s normal function, as interactions with surrounding amino acids play a critical role in protein stability and activity.

## 4. Discussion

The severity of COVID-19 symptoms varies widely among individuals, indicating the involvement of various genetic and environmental factors in disease progression and outcome [18]. Mitochondria, the vital organelles responsible for energy production within cells, play a critical role in the immune response and cellular homeostasis [19,20]. Specifically, alterations in mtDNA have been associated with increased susceptibility to various diseases, including infectious diseases [21]. Studies suggest that SARS-CoV-2 infection triggers intense inflammation and oxidative stress, causing damage to mtDNA, which is particularly vulnerable to mutations. Excessive ROS generated during infection can harm mtDNA, leading to mutations that impair mitochondrial function. Such mutations may contribute to disease severity, chronic symptoms, or post-viral syndromes like long COVID by affecting cellular energy production. In addition, altered mitochondrial dynamics due to SARS-CoV-2 infection result in increased cytokine production and cell death [22]. Therefore, understanding the mitochondrial dysfunction and any related variations behind this is essential for knowing the relationship with the disease [10].

In this work, *MT-CYB* and *MT-ATP6* genes of mtDNA were investigated. Cytochrome b, a key component of complex III in the electron transport chain, plays a major role in energy metabolism through oxidative phosphorylation [15]. It facilitates the electron transfer from ubiquinol to cytochrome c and utilizes the produced energy to transfer protons from the inside of the mitochondrial inner membrane to the outside. The ATP6 protein, a subunit of the electron transport chain complex V, is crucial for ATP generation [20]. It forms the channel of complex V and serves as the entry point for protons, which enter the matrix and use energy to produce ATP [20]. However, due to genetic defects, when the normal physiological condition of these complexes is disturbed, ATP synthesis gets lowered, and the ROS level increases. These create a disturbance in calcium homeostasis, resulting in oxidative stress [14]. Thus, any change to the *MT-CYB* or *MT-ATP6* gene that modifies these protein structures can lead to an alteration in ligand binding or translation rate. This impairment affects their ability to transport protons to the electron transport chain, thereby reducing ATP production. Ultimately, altered activities of mitochondrial ETC due to changes in any of its components lead to an impaired cellular energy state that could be one of the mechanisms leading to the pathophysiology of infectious diseases like COVID-19 [23].

In this study, the genetic alterations of the *MT-CYB* gene were identified and analyzed. Our analysis revealed a total of 9 variations within this mtDNA region from 14747–15887 bp that encodes cytochrome-b (Table 2). Out of these variants, synonymous SNPs m.15043G > A, m.15301G > A found in 80% and 70% of the patients, respectively, and m.14956T > C, m.15049C > T, m.15295C > T, and m.15346G > A showed less than 5% frequency in the patients. This finding is also similar to the frequency in healthy controls and reported global frequency (as per MITOMAP and gnomAD 3.1) (Tables 2 and 4).

On the other hand, nsSNPs m.14942A > C and m.15315C > T showed 20% and 3.33% frequency in the study population, whereas, in MITOMAP, gnomAD 3.1 database, the frequency was less than 0.4% (Table 4). Interestingly, m.14942A > C and m.15315C > T showed 0% frequency in healthy controls (Table 2). However, only one nsSNP m.15326A > G (causing amino acid alteration Thr194Ala) was found in 100% (n = 30) patients and 100% (n = 80) healthy controls.

When the association of the variants with the disease severity was assessed statistically, one variant m.14942A > C showed a significant association with the disease (Fig 1). Here, m.14942A > C was an nsSNP causing amino acid alteration Ile66Leu. To determine the pathogenicity, 7 different pathogenicity predictor tools were utilized due to their high accuracy and the fact that they employ diverse algorithms. Out of 7 pathogenicity predictor tools, 5 revealed m.14942A > C as having the most deleterious effects on protein structure and function (Fig 3). Additionally, the effect of this nsSNP on protein stability was assessed using 5 web-based tools and predicted to be destabilizing by all (Fig 5). Furthermore, this variant showed 10% relative surface accessibility (threshold ~ 25%) and was thus confirmed to be located in the buried region (S2 Fig). While buried residues are primarily involved in preserving the protein’s correct structure, residues on the protein’s surface are crucial in interactions with ligands and other proteins. By altering several crucial interactions, mutations in the protein’s exposed area can directly affect function. The properties of the changed amino acids caused by the nsSNPs were assessed based on the results of HOPE. Variant m.14942A > C was found to be located in a domain that is important for the activity and in contact with another domain that is also important for the activity (Table 5).

As the study aimed to investigate how mutations were associated with changes in three-dimensional (3D) protein structure and affected the structural integrity, 3D structures of wild-type and mutant proteins due to the m.14942A > C mutation were predicted and differentiated (Fig 7). The protein’s structure has been significantly altered due to the mutation, resulting in the loss of a hydrogen bond with the previous amino acid and the formation of new hydrogen bonds. These changes could impact its stability, function, and overall biological activity.

Secondly, the genetic alterations of the *MT-ATP6* gene were identified and analyzed. Our analysis revealed a total of 8 variations within this mtDNA region from 8527–9207 bp that encodes the ATP6 subunit (ATP synthase subunit ‘a’) (Table 3). Out of these variants, the most frequent 2 variants were m.8701A > G and m.8860A > G, found in 70% and 100% of the patients, respectively. This finding also almost matched the frequency in healthy controls and the reported global frequency. In addition to these, synonymous SNPs m.8911T > C, m.8928T > C, m.9003C > T, and m.9004C > T showed less than 15% frequency in the study population, whereas, in MITOMAP, gnomAD 3.1 database the frequency was less than 1% (Table 4). All of them were present in patients and healthy controls at almost an equal frequency. The other 2 nsSNPs, m.8744T > G and m.8794C > T, were found at frequencies of 23% and 3.33% in the patients, respectively. According to MITOMAP and gnomAD 3.1 databases, the global frequency of m.8744T > G was 0.002% and 0.004%, but for m.8794C > T, the frequency was 2.86% and 4.75%, respectively (Table 4).

When the association of the variants with the disease severity was assessed, m.8744T > G and m.8701A > G showed a significant association with the disease (Fig 2). m.8701A > G mutation was polymorphic according to a previous study. So, it can be hypothesized that the m.8744T > G mutation correlates with the disease. Here, m.8744T > G was an nsSNP causing amino acid alteration Val73Gly. To determine pathogenicity, 7 different pathogenicity predictor tools were utilized because these tools have high accuracy and employ diverse algorithms. Out of 7 pathogenicity predictor tools, 4 tools revealed m.8744T > G as having the most deleterious effects on protein structure and function (Fig 4). Additionally, the effect of nsSNPs on protein stability was assessed using 5 web-based tools and predicted to be destabilizing by all (Fig 6). The ΔΔG value was used to determine the stability of proteins. Furthermore, mutant amino acids encoded by variant m.8744T > G showed 24% relative surface accessibility (threshold ~ 25%) and were thus confirmed to be located in the buried region (S3 Fig). Moreover, according to the HOPE tool (which assesses properties of the changed amino acids due to nsSNPs), this mutation introduces a smaller mutant residue, and the wild-type residue is more hydrophobic than the mutant residue (Table 5). These differences in size and hydrophobicity can affect the hydrophobic interactions with the membrane lipids. Hydrophobicity is sometimes important for multimerization; therefore, this mutation might impact the multimer interactions by introducing a glycine at this site. Glycines are highly flexible and could disrupt the necessary rigidity of the protein at this location. To associate mutation with changes in 3D protein structure and impact on the structural integrity, 3D structures of wild-type and mutant protein due to the m.8744T > G mutation were predicted and differentiated (Fig 8). This mutation significantly impacts protein interactions with surrounding amino acids, resulting in a decline in contacts and loss of hydrogen bonds with previous amino acids. This mutation could disrupt the protein’s normal function, as interactions with amino acids are crucial for protein stability and activity.

This study has some limitations. Functional validation experiments of the predicted variants were not performed due to limitations of resources and scope. Additionally, the sample size was relatively small, which may limit the statistical power of the findings. However, other researchers with appropriate resources can build upon this work.

In conclusion, this study identified one novel mtDNA mutation, along with several relatively common mutations in the *MT-CYB* and *MT-ATP6* genes among COVID-19 patients. As mtDNA mutations are commonly observed in COVID-19 patients, it indicates that SARS-CoV-2 may alter the host’s genetic material, particularly in mtDNA. While SARS-CoV-2 is an RNA virus that does not directly integrate into the host genome, the impact of the infection on cellular stress, inflammation, and oxidative damage can increase the risk of genetic alterations. Besides, from the above observations, it can be assumed that there may be a correlation between the m.14942A > C mutation in the *MT-CYB* gene as well as the m.8744T > G mutation in the *MT-ATP6* gene with the clinical symptoms of COVID-19. The results of this study will help better understand the links among SARS-CoV-2 infection, mtDNA mutations in these two genes, and mitochondrial dysfunction. Moreover, this study may serve as a valuable reference for further studies, as future work should validate these findings in larger, diverse populations.

## Supporting information

S1 Table Demographic information about COVID-19 patients and healthy individuals.
(DOCX)

S1 Fig Representative chromatograms of *MT-CYB* and *MT-ATP6* sequences of some samples aligned with the revised Cambridge Reference Sequence (rCRS).
(DOCX)

S2 Fig A box and whisker plot representing the surface accessibility of each of the amino acid positions that were altered as a consequence of the nsSNPs.
(DOCX)

S3 Fig A box and whisker plot representing the surface accessibility of each of the amino acid positions that were altered as a consequence of the nsSNPs.
(DOCX)
