# Supplementary material for: Evaluating the MT-CYB and MT-ATP6 variations in COVID-19 patients: A case-control study
Source: PLoS One. 2025 Aug 21;20(8):e0329866. doi: 10.1371/journal.pone.0329866 (PMC12370035; doi:10.1371/journal.pone.0329866)
Supplement: S1 Table — Demographic information about COVID-19 patients and healthy individuals. (DOCX) [file pone.0329866.s001.docx]

**S1 Table. Demographic information about COVID-19 patients and healthy individuals.**

| Variable | Case  (n = 30 patients) | Control  (n = 80 healthy individuals) | P-value |
| --- | --- | --- | --- |
| Age (years) mean ± SD* | 37.77 ± 13.92 | 31.71 ± 12.05 | 0.041 |
| Sex |  |  |  |
| Male | 63.33% | 51.25% | 0.297 |
| Female | 36.67% | 48.75% |  |
