# Supplementary material for: Evaluating the MT-CYB and MT-ATP6 variations in COVID-19 patients: A case-control study
Source: PLoS One. 2025 Aug 21;20(8):e0329866. doi: 10.1371/journal.pone.0329866 (PMC12370035; doi:10.1371/journal.pone.0329866)
Supplement: S1 Fig — Representative chromatograms of MT-CYB and MT-ATP6 sequences of some samples aligned with the revised Cambridge Reference Sequence (rCRS). (DOCX) [file pone.0329866.s002.docx]

**B**

**A**

**S1 Fig. Representative chromatograms of *MT-CYB* and *MT-ATP6* sequences of some samples aligned with the revised Cambridge Reference Sequence (rCRS).** (A) Partial chromatogram shows novel mutation m.14942A>C in *MT-CYB* gene. (B) Partial chromatogram illustrates mutation m.8744T>G in the *MT-ATP6* gene.
