# Supplementary material for: Evaluating the MT-CYB and MT-ATP6 variations in COVID-19 patients: A case-control study
Source: PLoS One. 2025 Aug 21;20(8):e0329866. doi: 10.1371/journal.pone.0329866 (PMC12370035; doi:10.1371/journal.pone.0329866)
Supplement: S2 Fig — A box and whisker plot representing the surface accessibility of each of the amino acid positions that were altered as a consequence of the nsSNPs. (DOCX) [file pone.0329866.s003.docx]

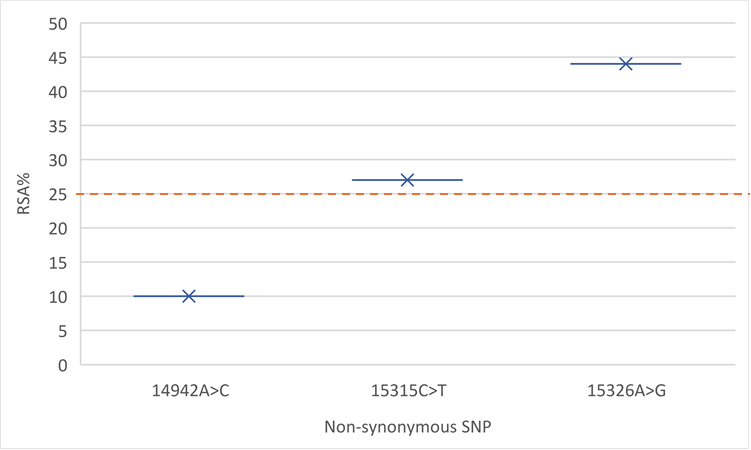


**S2 Fig. A box and whisker plot representing the surface accessibility of each of the amino acid positions that were altered as a consequence of the nsSNPs**. Here, the nsSNPs found in the n *MT-CYB* gene, causing the amino acid change, are annotated with each bar at the x-axis. The y-axis indicates the relative surface accessibility (RSA) value. The threshold level of RSA is 25% and nsSNPs exceeding the threshold are considered surface exposed.
